# Omission of Risk in Vitamin A–Related Dermatologic Instagram Posts: A Growing Concern in an Unregulated Digital Landscape

**DOI:** 10.2196/77504

**Published:** 2025-09-12

**Authors:** Robert John Vanaria, Michael Povelaitis, Riya Khokhal, Shannon Meledathu, Claudia Green, Robin Ashinoff, Mariela Mitre

**Affiliations:** 1 Hackensack Meridian School of Medicine, 123 Metro Blvd, Nutley, NJ, 07110, United States; 2 Hackensack University Medical Center, 360 Essex St Suite, #201, Hackensack, NJ, 07601, United States, 1 551 996 2000

**Keywords:** social media, Instagram, retinoid, retinol, risk, regulation

## Abstract

We reviewed 30 of the top-viewed Instagram videos using the hashtags #retinoid and #retinol to assess reliability using the DISCERN instrument. Dermatologists produced more accurate content than laypeople, though important details such as treatment risks were often omitted. Our findings highlight the need for health professionals to balance accessibility with accuracy to provide trustworthy dermatologic information on social media.

## Introduction

Social media has become a daily source of connectivity, entertainment, and health information across all age groups. It has transformed how health knowledge is shared and consumed. Often, such content is delivered under the disclaimer “this post is not medical advice,” even as it implies otherwise. A lack of platform regulation allows individuals without medical training to share health-related information or personal experiences, often omitting context critical to patient understanding and safety. To increase engagement, even health care professionals–including dermatologists–have adapted their content into brief, attention-grabbing formats. This shift toward short-form, simplified videos often sacrifices educational depth, reducing complex topics to superficial overviews. The emphasis on visual appeal may further prioritize style over substance. Past studies evaluating social media health content often conclude that improved quality is essential for patient safety [1-3].

## Methods

To assess the reliability of dermatologic information on Instagram, we analyzed the top 30 most-viewed videos under the hashtags #retinoid OR #retinol, each with over 50,000 views. There were no duplicate videos. The decision to include 30 videos was based on previous publications in the field that included a similar or smaller number of videos for their statistical analysis. Content creators were categorized as dermatologists, nondermatology physicians (other MDs/DOs), or laypeople. Videos were excluded if the creator’s qualifications could not be verified. Four independent reviewers with dermatology training scored each video using the DISCERN instrument, a validated tool for assessing consumer health information quality [4]. Because not all consumers read further into social media posts, we did not include captions, descriptions, and on-screen text in the DISCERN tool analysis. Median and mean scores for each group were calculated. A 1-way ANOVA was used to test for group differences, with post hoc Tukey analysis used for pairwise comparisons (significance set at *P*<.05).

## Results

**Figure 1.**
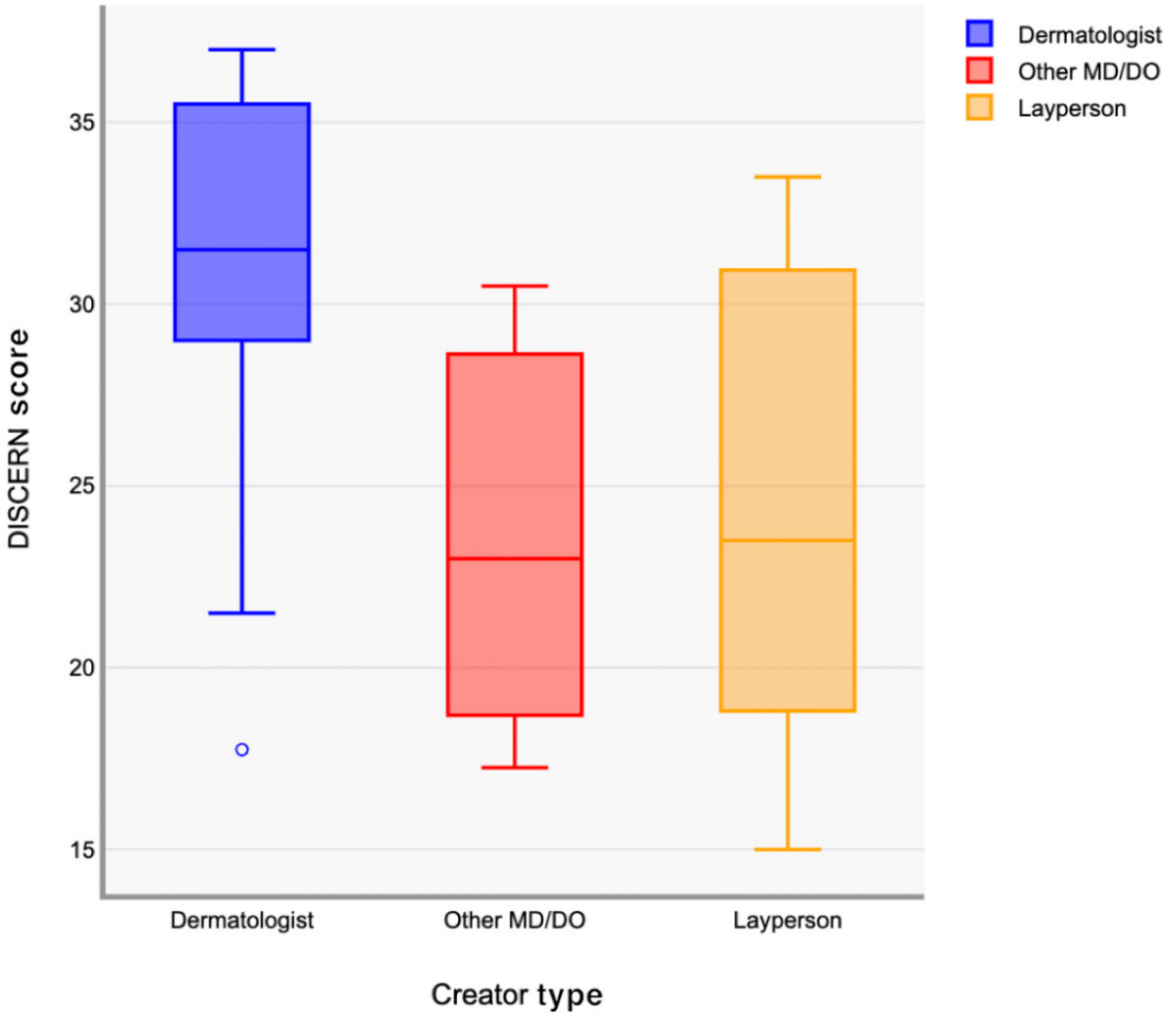
Boxplot showing differences in average DISCERN scores across different creator categories.

The breakdown of videos was as follows: 10 dermatologist-created, 3 other physician–created, and 17 layperson-created. We found a significant difference in DISCERN scores by creator type (*P*=.04). Post hoc analysis showed dermatologist-created videos scored significantly higher than those by laypeople (*P*=.049). Differences between dermatologists and nondermatology professionals (*P*=.09) and between nondermatology professionals and laypeople (*P*=.98) were not significant. The intraclass correlation coefficient among reviewers was 0.958, indicating excellent concordance. Results are shown in Figure 1.

## Discussion

### Principal Findings

Although dermatologist videos were higher in quality, even these showed major gaps. One commonly missed DISCERN criterion was question 11: “Does it describe the risks of each treatment?” This was underreported across all groups. This omission is concerning, as topical retinoids, though effective, are associated with irritation, dryness, and photosensitivity [5,6]. If these risks are not clearly explained, misuse or discontinuation may occur.

Interestingly, dermatologists outperformed other physicians, suggesting that specialty-specific expertise matters when communicating dermatologic information. Despite general medical training, nondermatology professionals may lack the nuanced understanding necessary to convey accurate skincare content.

Social media wields enormous influence on public health decisions. While creators may aim to educate, oversimplified or incomplete content can mislead. Our findings highlight the importance of evidence-based communication, especially when addressing topics outside one’s specialty. Clear presentation of risks, benefits, and limitations is essential to support informed, safe choices.

This study has limitations. Our analysis was limited to 30 videos, which may not capture the full range of retinoid-related content on Instagram. The DISCERN tool, though validated, was designed for written material and may not fully assess video nuances. Lastly, classification of creators was based on publicly available data and could be subject to misidentification.

### Conclusion

Dermatologists and other health professionals must remain vigilant in sharing balanced, accurate information on social media. Improving the quality and transparency of content can help transform platforms like Instagram into trusted resources for skin health rather than sources of misinformation. Additionally, content regulation by social media companies is essential for protecting patients, as unregulated medical information can cause significant harm to patients.
